# Walter Edward Dandy (1886–1946)

**DOI:** 10.1007/s00415-025-13156-3

**Published:** 2025-06-01

**Authors:** Alisha Huang, Maeve C. Lucas, Mariam M. Yousuf, Lilia Kazerooni, Jonathan D. Santoro

**Affiliations:** 1 University of Southern California, Los Angeles, CA USA; 2 Division of Neurology, Department of Pediatrics, Children’s Hospital Los Angeles, 4650 Sunset Blvd, Mailstop 82, Los Angeles, CA 90027 USA; 3 Department of Neurology, Keck School of Medicine of the University of Southern California, Los Angeles, CA USA

**Fig. 1:**
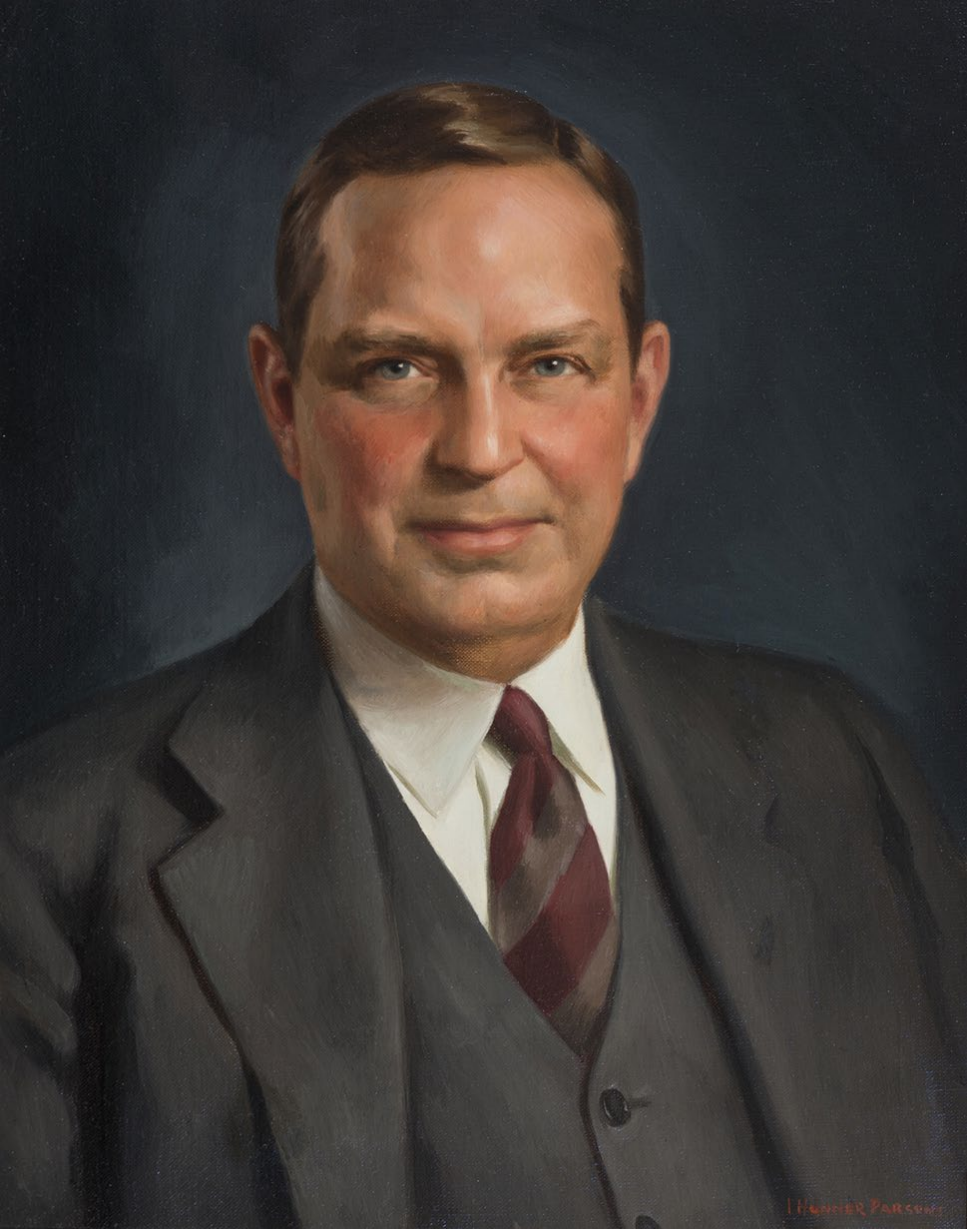
Walter Edward Dandy (1886–1946). Oil on canvas painting by Isabella Hunner Parsons (1903–1968). Reproduced with permission courtesy of The Alan Mason Chesney Medical Archives of The Johns Hopkins Medical Institutions. (https://medicalarchives.jhmi.edu/portrait/dandy-walter-edward2/)

Walter Edward Dandy (Fig. 1) was born on April 6, 1886, in Sedalia, Missouri, the only child of immigrant parents: a British father, John, fireman turned locomotive engineer, and an Irish mother, Rachel, a seamstress [1]. Graduating from Sedalia High School one year early as valedictorian in 1903, Dandy went on to attend the University of Missouri, where his enthusiasm for biology and medicine burgeoned [2]. As an undergraduate, he became involved in laboratory research and worked as an assistant to Winterton C. Curtis in the zoology laboratory [2].

Dandy enrolled in 1907 at Johns Hopkins School of Medicine as a sophomore, guided by Curtis and by William Osler [1, 3]. Anatomy attracted Dandy, who eventually worked under the pioneer embryologist–anatomist Franklin P. Mall at Hopkins [3]. As a trainee adept at dissection and drawing, Dandy was assigned by Mall the study and representation of the youngest human embryo preserved in his collection, which was subsequently published and referenced as “The Dandy embryo” in the medical literature [2].

Upon receiving his MD in 1910, Dandy trained in surgery under William Halsted. During his surgical training, he also carried out research as a clinical assistant to Harvey W. Cushing at the Hunterian Surgical Laboratory of Experimental Science [3]. Under Cushing’s guidance, Dandy explored hydrocephalus in collaboration with Kenneth D. Blackfan, in addition to investigating the innervation and vasculature of the pituitary of dogs, cats, and other animals for six years [2]. Dandy successfully carried out numerous operations that his mentor considered preposterous, including treating an epilepsy patient surgically in 1912, something that Cushing had never succeeded in doing previously [4]. Henceforth, a taut yet fascinating master-apprentice feud between the two minds transpired, which persisted for the remainder of their careers. Dandy eventually performed over 2000 posterior fossa operations and quadrupled the number of pituitary operation performed by Cushing [3, 4]. Notably beyond the volume of Dandy’s surgical interventions is that his innovative approaches led to marked reductions in morbidity and mortality of complex surgical interventions in the posterior fossa and cerebellopontine angle [5].

After Cushing’s decision to leave Baltimore, Dandy received a temporary appointment at Hopkins by the Hospital administrator, Winford Smith, and was promoted to assistant resident in general surgery by Halsted in 1914 [2]. In that year, Blackfan and Dandy identified the Dandy–Walker syndrome, a rare congenital neurodevelopmental malformation mainly affecting the cerebellum, and characterized by hypoplasia of the vermis, cystic dilation of the fourth ventricle, and an enlarged posterior fossa [5].

From his first operation of a patient with hydrocephalus in January 1915 until his last operation in 1946, Dandy made important strides in the surgical management of hydrocephalus via choroid plexus cauterization, a procedure globally accepted today. He also pioneered the technique of perforation of the floor of the third ventricle to create a bypass for cerebrospinal fluid, a modern-day endoscopic third ventriculostomy, with open surgery [5, 6]. Furthermore, Dandy provided a basis for classifying the subtypes of hydrocephalus [6] and for the diagnosis of hydrocephalus and localization of intracranial tumors by X-ray ventriculography and pneumoencephalography, which Cushing looked down upon, and which would later lead to Dandy’s multiple Nobel Prize nominations in the 1930s [3, 4, 7]. In doing so, he became an early pioneer in the field of pediatric neurosurgery. Intracranial aneurysm therapy was pivotally affected by Dandy, who became the first person to employ a silver clip to surgically treat and excise an internal carotid artery aneurysm in 1937—eight years after his middle cerebral aneurysm operations had yielded disappointing results [5].

In 1928, Dandy was credited for introducing the groundbreaking technique of anatomic hemispherectomy to treat seemingly incurable glioblastomas and to remove unilateral brain neoplasms [8]. Dandy’s operation on five patients with right hemisphere glioma involved initially ligating the main veins as well as the middle and anterior cerebral arteries at the carotid bifurcation and subsequently removing the cerebral hemisphere fragment at a time [8]. Specifically, Dandy sectioned the frontal lobe and proceeded to split the corpus callosum, through an incision of the internal capsule [8]. By removing the entire right cerebral hemisphere from each of the five patients, Dandy aimed at eliminating the hemiplegia-related tumor without the mental impairment of a right-handed person [5, 8]. As detailed in Dandy’s preliminary report of 1928, while some patients passed away shortly after surgery, one lived for 3.5 years post-operatively, even regaining certain abilities, such as partial knee and thigh extension and flexion [5]. Indeed, Dandy set the precedent for successful, safer, and seizure-free hemispherectomy operation variants to come, including functional or disconnection hemispherectomy, which was developed by Theodore Brown Rasmussen in 1974, and is now used as the basis for a successful treatment in a plethora of nervous diseases, including Rasmussen encephalitis, Sturge–Weber syndrome, hemimegalencephaly, and hemispheric epilepsy [8]. His contributions to the treatment of neoplasia were not singularly limited to the brain as Dandy also led groundbreaking work on surgical management of tumors in the spinal cord. His work on intramedullary tumors of the spine, a particularly challenging tumor type to treat in the era in which he operated, are particularly notable [9].

Bold, brilliant, and boundless, Walter Edward Dandy was an ardent neurosurgeon and an artist, who modernized and illuminated the field of neurosurgery. Throughout his professional career, Dandy published five books, including the classic text *Surgery of the Brain*, and authored more than 160 papers [6]. On April 1, 1946, a pain in the chest forced Dandy to suspend work with his youngest child Margaret, in the garden [10]. Following the diagnosis of a heart attack, Dandy was able to recover and return home to his wife Sadie Estelle Martin and children—but not for long [10]. Dandy passed away several weeks later, on April 19, 1946, from a second heart attack at the institute where his entire illustrious neurological career commenced: the Johns Hopkins Hospital [10]. 
